# Replication competence of virions induced from CD4+ lymphocytes latently infected with HIV

**DOI:** 10.1186/s12977-019-0466-1

**Published:** 2019-02-15

**Authors:** Douglas D. Richman, Karissa Huang, Steven M. Lada, Xiaoying Sun, Sonia Jain, Marta Massanella, Bryson Menke

**Affiliations:** 1 San Diego Veterans Affairs Healthcare System, San Diego, CA USA; 2 University of California San Diego, La Jolla, CA USA; 3 Université de Montréal, Montreal, QC Canada; 4 Present Address: University of Colorado Denver School of Medicine, Aurora, CO USA; 5 Present Address: University of California, Irvine, CA USA

**Keywords:** HIV, Latency, Quantitative viral outgrowth, Reservoir

## Abstract

Latently infected CD4 lymphocytes preclude cure of HIV infection, even with the most effective antiretroviral therapy. The replication competent latent HIV reservoir has been quantified with the terminal dilution quantitative viral outgrowth assay, which induces virus propagation in CD4^+^ T cell culture supernatants following cellular activation. Efforts to improve the sensitivity of this inefficient assay have introduced more sensitive p24 ELISA and RNA PCR based endpoints, but these more sensitive endpoints have raised the question whether they are measuring induced replication competent or defective virions. Here we performed parallel terminal dilution assays with CD4 lymphocytes from subjects effectively treated with antiretroviral therapy. An HIV integrase inhibitor was incorporated into one set of parallel cultures to compare the frequency of cells that can be induced to produce virions to those that produce virus that can propagate and amplify with co-culture in permissive cells. The majority of cells that can be induced to generate virus particles are producing replication competent virus, thus justifying more sensitive and faster assays of this reservoir.

**Electronic supplementary material:**

The online version of this article (10.1186/s12977-019-0466-1) contains supplementary material, which is available to authorized users.

HIV DNA integrated into host cell DNA of resting CD4^+^ T lymphocytes can be induced to produce infectious virus upon cellular activation. Effective inhibition of virus replication by antiretroviral therapy (ART) of HIV-infected individuals cannot cure HIV infection because of this latent HIV reservoir [1–3]. The quantitative viral outgrowth assay (QVOA) measures the frequency of latently infected cells by expanding replication competent virus after activation of terminally diluted CD4 lymphocytes, with most assays using resting CD4 cells [4]. Therefore, in this assay, replication competent virus must propagate in uninfected cells, resulting in ongoing amplification of viral protein (p24) or RNA. Approximately one in a million CD4^+^ T cells is latently infected using standard QVOA assays; however, intact replication competent proviruses may exceed those that can are measured with the standard QVOA assay by on average 64-fold in one study [5] and 25 to 27-fold in a subsequent study [6, 7]. An important question is how much this discrepancy is attributable either to provirus integrated into sites not amenable to reactivation or to the inefficiencies of the conditions of in vitro assays compared to in vivo conditions. Repeated reactivation of cells can disclose additionally replication competent proviruses, but do not increase QVOA values more than twofold [8].

Recently efforts to improve QVOA sensitivity have been implemented by replacing the standard HIV p24 ELISA to detect the production of virus induced by activated cells in culture supernatants using either a PCR assay for cell-free HIV RNA or a more sensitive digital ELISA with increased sensitivity for HIV p24 (at femtogram levels) [7, 9, 10]. The main concern with these assays is that their sensitivity for virion components in the culture supernatant may also detect low levels of induced extracellular virions that are not replication competent. To ascertain how much virion production, as measured with PCR for cell-free HIV RNA, represented the induction of replication competent virus from latently infected cells, we performed parallel QVOA assays in the presence or absence of the integrase inhibitor raltegravir, and measured virus production frequently over the course of 2 weeks. These assays were performed with the CD4^+^ T cells from 5 subjects effectively treated with ART for more than 1 year, as detailed in Methods. This approach permitted a sense of the kinetics of virion induction without ongoing propagation in the presence of raltegravir compared to a measurement of the frequency of latently infected cells that were replication competent in the absence of raltegravir.

The time course of production and persistence of supernatant virions in the presence of raltegravir, which prevents ongoing viral propagation can be seen in the left panels of Fig. 1a. The time course of production and persistence of supernatant virions in the absence of raltegravir, thus permitting viral propagation, can be seen in the right panels of Fig. 1a. In the assays shown there are 6 replicates at each of the threefold dilutions starting with 1 million cells per well in dilution 1.

**Fig. 1:**
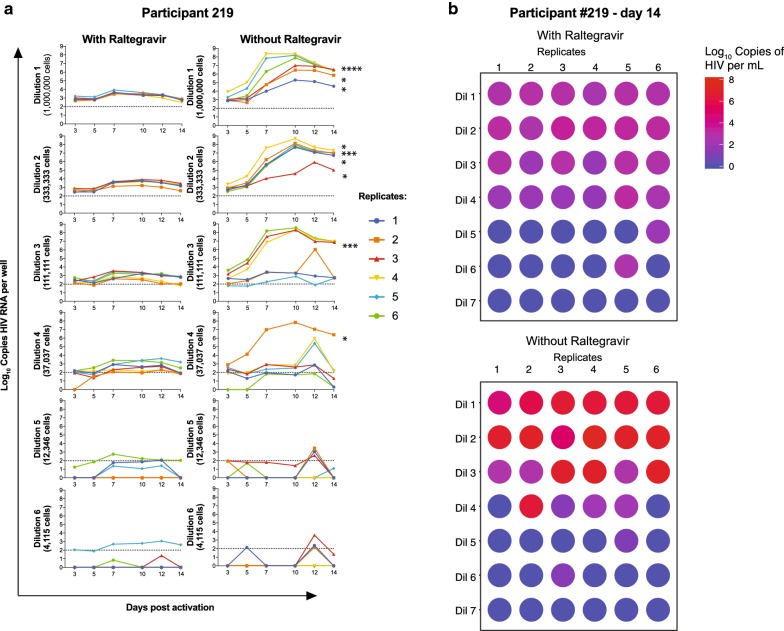
Parallel QVOA results in the presence or absence of raltegravir with CD4 lymphocytes from subject 219. Six replicates were performed for each serial threefold dilution of CD4 cells in the QVOA assay with (**a**, left row) or without (**a** right row) raltegravir in the culture medium throughout the assay shown on a log_10_ scale. Culture supernatants were assayed for HIV RNA by real time (RT)-PCR. The asterisks on the lines of the replicates without raltegravir on day 14 indicate values for each well without raltegravir that exceeded the mean plus 5 standard deviations of the 6 replicates performed with raltegravir. **b** Displays the same data for day 14 to indicate visually that virions are induced in the presence of raltegravir (upper figure) and that amplification occurs in almost as many wells (lower figure)

As can be seen in Fig. 1, cells can be induced to produce a substantial number of virions even when ongoing replication is inhibited with raltegravir. Values tend to diminish with each dilution as the number of cells with HIV proviruses that can be induced become fewer. It is not possible to clearly ascertain the number of cells carrying proviruses in each well that contribute to cell free HIV RNA, although the likelihood of just a single cell contributing increases with each dilution of input CD4+ cell number. In the presence of raltegravir virions are usually detectable 3 days after induction; however, in general either new cells begin to produce virus after 3 days or the cells progressively increase the production of virus by day 7 after activation. Virion production persists and remains in the supernatant even while the CD4+ MOLT-4/CCR5 cells propagate in the culture wells. Continuing production is likely, because virions at a level of 1000 copies of HIV RNA when placed in a cell-free well on day 0 do not persist and are no longer detectable after 1 or 2 days (data not shown).

Since it is not possible to measure the induction and propagation of HIV after activation in the same cells both with and without raltegravir, parallel experiments were performed with cells from the same participant. The results of these comparisons performed as shown in Fig. 1 were performed in 4 other study subjects (Additional file 1: Fig. S1). Different definitions of replication competence in the culture wells without raltegravir were then explored to ascertain a sense of the difference between total induced virions (with raltegravir) compared to the individual culture wells without raltegravir that fulfill a definition of propagation of replication competent virus. Two different criteria of amplification in the absence of the inhibitor were examined in Table 1.

**Table 1:** Determinations of infectious units per million (IUPM) cells with or without raltegravir using different criteria

Study subject	With raltegravir day 14		Without raltegravir day 14		Amplification day 14 without raltegravir		Amplification AUC without raltegravir	
	IUPM	95% CI	IUPM	95% CI	IUPM	95% CI	IUPM	95% CI
197	0.7	0.3–1.8	7.8	3.8–15.9	5.7	2.9–11.2	4.8	2.4–9.6
215	9.9	5.0–19.5	18.3	9.5–35.2	6.5	3.5–12.2	1.5	0.8–2.8
216	0.1	0.3–0.3	1.1	0.5–2.2	1.2	0.6–2.5	1.2	0.6–2.5
217	1.1	0.5–2.2	0.5	0.2–1.4	0.5	0.2–1.4	0.9	0.4–1.9
219	15.7	8.1–30.5	18.7	9.2–38.2	7.1	3.5–14.2	14.4	7.6–27.5

IUPM were calculated as described in Methods. Column 1: Subject number. Column 2: IUPM with a positive well defined as containing > 100 copies HIV RNA per ml on day 14 in the presence of raltegravir. Column 3: IUPM with a positive well defined as containing > 100 copies HIV RNA per ml on day 14 in the absence of raltegravir. Two criteria were used to define amplification (replication competence) in the absence of raltegravir: Column 4: A well was defined as positive on day 14 without raltegravir when the value was greater than the mean plus 5 SD of the 6 wells in the presence of raltegravir. Column 5: The criterion for a positive well assessing amplification by AUC without raltegravir used the mean and SD of the area under the curve for the 2 weeks of measurements for all six wells in that dilution in the presence of raltegravir

The results of these experiments indicated that the detection of induced virion production from latently HIV-infected cells is a close reflection of the number of cells harboring replication competent virus, although for most subjects on average virus amplification is lower than the number of cells generating virions on most days of culture (Fig. 2). The values for IUPM with the QVOA without raltegravir on day 14 ranged between 1 and 11-fold higher than those in the presence of raltegravir (Table 1). When the 2 rigorous criteria for amplification of replication competent virus were examined, the IUPM calculations were not substantially diminished. The results from this study indicated that there are more CD4^+^ T cells that produce virions after activation than those that produce replication competent virus; nevertheless; by examining a rigorous criterion for amplification of replication competence at day 14, virions that are induced by activation of CD4^+^ T cells are in general replication competent. A recent publication using the digital p24 assay in the QVOA from 1 subject showed 8 induced cells generated viral antigen that amplified and 9 that did not, consistent with the results from this study [11].

**Fig. 2:**
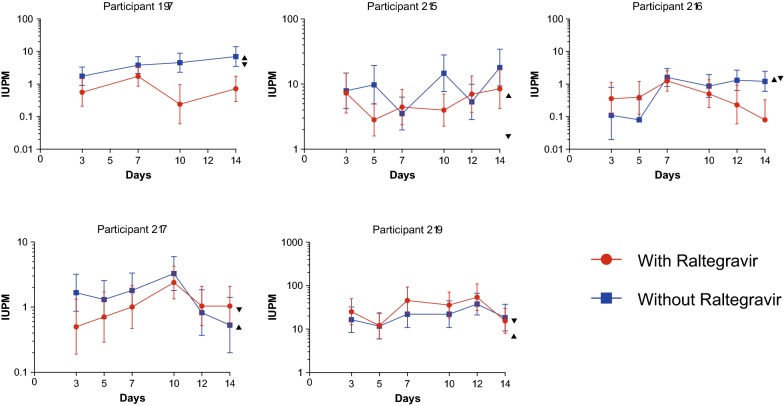
IUPM values with or without raltegravir for each day of culture for the 5 study subjects. IUPM values (with 95% CI) are graphed for each study subject for each day of supernatant collection. Positive values were defined as > 100 copies HIV RNA/mL. Also indicated for each subject with the day 14 results are the IUPM values using the 2 criteria for amplification (replication competence): day 14 criterion (▲) and AUC criterion (▼)

There are a number of sources of imprecision and variability in these measurements. First parallel assays needed to be performed, because there is no way to have an individual well with the same cells be both exposed and not exposed to raltegravir. Second, as indicated with the 95% confidence intervals, there is inherent imprecision in any terminal dilution assay that contains a realistic number of dilutions and replicates per dilution, considering the cells available from study subjects. Third, it is well established that after prolonged ART, the proportion of clonally expanded CD4 cells with integrated HIV DNA progressively increases [8, 12, 13]. The composition of these clones, which can be either competent or defective proviruses will certainly vary between subjects. Nevertheless, the detection of HIV antigen or RNA can represent a relatively accurate surrogate measurement of the true replication competent latent reservoir. A 3-day assay of virion induction (cell-free RNA) was recently shown to produce measurements within twofold of the increased sensitivity of using HIV RNA to measure propagation of HIV after a 14-day QVOA as used in these experiments [7, 9]. The replication competence of the virions in that study was not determined, although the values generated were within twofold of the IUPM values. In the current study parallel assays with and without raltegravir confirmed that induction of virions generates similar values to those determined to be replication competent. The possibility is thus raised that a shorter and easier measurement of induced virus may prove of similar utility; however, validation will require comparing these assays when an effective intervention becomes available.

Table 1 indicates that measuring statistically significant amplification without raltegravir generates IUPM values in a similar range to the IUPM values using RNA produced in the presence of raltegravir. Moreover, the detection of cell-free virions in these assays that do not propagate may be important targets for candidate HIV “cure” strategies. First, failure to demonstrate propagation of a virion in a culture well may not reflect the capacity of that virion to ignite ongoing replication in the lymphoid tissue of an infected individual where conditions and cell density may be much more conducive to replication [14]. Second, even defective virions, which do compose some of the low-level viremia during effective ART, may still contribute to the immune activation and the non-AIDS related complications of well treated HIV infection. In summary, a high proportion and often the majority of cells that can be induced to generate virions upon activation are producing replication competent virions. This information should support the rationale to utilize more sensitive and shorter assays to measure the replication competent latent HIV reservoir.

## Methods

### Participant samples

Peripheral blood was obtained from HIV infected donors and processed for peripheral blood mononuclear cells (PBMC) to prepare CD4^+^ T lymphocytes. The donors had been chronically HIV-infected with baseline viral loads between 40,000 and 500,000 copies HIV RNA/mL plasma before receiving ART and being suppressed below 50 copies for all measurements for at least 1 year. All had > 500 CD4 cells/μL blood at the time of donation, which followed a UCSD Institutional Review Board informed written consent. PBMC were isolated using density gradient centrifugation from whole blood. Total CD4+ T cells were enriched from PBMC by negative selection using Stem Cell Technologies EasySep CD4 Enrichment kit previously described [7].

### Modified quantitative viral outgrowth assay (mQVOA) and Induced cell free (cf)-HIV RNA assays

As previously described in detail, enriched CD4+ T cells were serially diluted in a 24-well plate coated with anti-CD3 (Clone SK7) and anti-CD28 (Clone CD28.2) monoclonal antibodies (both from BD Biosciences) [7]. We have elected to measure total negatively selected CD4 lymphocytes rather than just resting cells, since in subjects well suppressed on ART all HIV-infected CD4 cells represent the reservoir that must be addressed with eradication strategies for cure. Six serial threefold dilutions were performed with starting concentrations of 1 × 10^6^ cells/well and with 6 replicates for each dilution. The concentration of raltegravir used throughout the QVOA assay was 250 nG/mL, which is 40 times the human serum adjusted IC_95_ of 31 nM.

After 2 days of stimulation, 200,000 MOLT-4/CCR5 cells (NIH AIDS Reagents program) were added to each cell culture well (day 0). Cell cultures were split twice weekly; half of cell culture supernatants (500 μL) were collected for analysis at days 3, 5, 7, 10, 12 and 14, except for CARE 197 with collections on days 3, 7, 10 and 14. Of note extending the assay beyond 14 days rarely if ever adds a positive well, as has been shown with the original description of the use of the MOLT-4/CCR5 cells [15]. Supernatants were spun at 300 g for 10 min and frozen at − 80 °C until use. HIV-specific magnetic beads were used to extract cell free-RNA from supernatants (Aptima HIV-1 kit, Hologic Incorporated). Briefly, supernatants were incubated with 400 μl of Target Capture Reagent (TCR, containing HIV-specific magnetic beads) for 7 min at 80 °C, 30 min at 60 °C and 22 min at 25 °C (a gift from Hologic, Inc.). After incubation, the magnetic particles were concentrated using a KingFisher instrument (Life Technologies). Extracted HIV RNA was then detected using One-step RT-PCR (Applied Biosystems) for detection of pol (integrase) [16]. The number of wells positive for HIV RNA was determined using the criteria described in Fig. 1, and the maximum likelihood method was applied to determine Infectious Units Per Million (IUPM) with the Infection Frequency Calculator (http://silicianolab.johnshopkins.edu/) [17].

### Statistical analysis

Data were analyzed on a log_10_ scale. Descriptive analyses including area under the curve (AUC) were used to summarize the data. Replicates without raltegravir were individually compared to the mean plus 5 standard deviations of the 6 replicates performed with raltegravir. The criterion for a positive well assessing amplification on day 14 used the mean and SD for all six wells in that dilution in the presence of raltegravir. A positive well without raltegravir was defined as greater than the mean plus 5 SD in the presence of raltegravir. The criterion for a positive well assessing amplification by AUC without raltegravir used the mean and SD of the area under the curve for the 2 weeks of measurements for all six wells in that dilution in the presence of raltegravir. A positive well without raltegravir was defined as greater than the mean plus 5 SD of the area under the curve in the presence of raltegravir. Statistical analyses were performed using Prism 7.0 and R software (version 3.3.2, http://www.Rproject.org/) [18].

## Additional file

**Additional file 1: Fig. S1.** Parallel QVOA results in the presence or absence of raltegravir with CD4 lymphocytes from 4 additional subjects. Six replicates were performed for each serial threefold dilution of CD4 cells in the QVOA assay with (**a**–**d** left column) or without (**a**–**d** right column) raltegravir in the culture medium throughout the assay shown in log_10_ scale. Culture supernatants were assayed for HIV RNA by real time (RT)-PCR. The asterisks on the lines of the replicates without raltegravir on day 14 indicate values for each well without raltegravir that exceeded the mean plus 5 standard deviations of the 6 replicates performed with raltegravir. Columns **e**–**h** display the same data for day 14 to indicate visually that virions are induced in the presence of raltegravir (upper figure) and that amplification occurs in almost as many wells (lower figure).

## Abbreviations

ART: antiretroviral therapy
AUC: area under the curve
CI: confidence interval
DNA: deoxyribonucleic acid
ELISA: enzyme linked immunosorbent assay
HIV: human immunodeficiency virus
IUPM: infectious units per million
PBMC: peripheral blood mononuclear cells
PCR: polymerase chain reaction
QVOA: quantitative viral outgrowth assay
RNA: ribonucleic acid
SD: standard deviation

## References

[1] Wong JK (1997). Recovery of replication-competent HIV despite prolonged suppression of plasma viremia. Science.

[2] Finzi D (1997). Identification of a reservoir for HIV-1 in patients on highly active antiretroviral therapy. Science.

[3] Chun TW (1997). Presence of an inducible HIV-1 latent reservoir during highly active antiretroviral therapy. PNAS.

[4] Massanella M, Richman DD (2016). Measuring the latent reservoir in vivo. J Clin Investig.

[5] Ho YC (2013). Replication-competent noninduced proviruses in the latent reservoir increase barrier to HIV-1 cure. Cell.

[6] Bruner KM (2016). Defective proviruses rapidly accumulate during acute HIV-1 infection. Nat Med.

[7] Massanella M (2018). Improved assays to measure and characterize the inducible HIV reservoir. eBioMedicine.

[8] Hosmane NN (2017). Proliferation of latently infected CD4^+^ T cells carrying replication-competent HIV-1: potential role in latent reservoir dynamics. J Exp Med.

[9] Rosenbloom DIS (2019). Assessing intra-lab precision and inter-lab repeatability of outgrowth assays of HIV-1 latent reservoir size. PLoS Comput Biol.

[10] Passaes CPB (2017). Ultrasensitive HIV-1 p24 assay detects single infected cells and differences in reservoir induction by latency reversal agents. J Virol.

[11] Bertagnolli LN (2018). The role of CD32 during HIV-1 infection. Nature.

[12] Wagner TA (2013). An increasing proportion of monotypic HIV-1 DNA sequences during antiretroviral treatment suggests proliferation of HIV-infected cells. J Virol.

[13] Reeves DB (2018). A majority of HIV persistence during antiretroviral therapy is due to infected cell proliferation. Nat Commun.

[14] Strain MC, Richman DD, Wong JK, Levine H (2002). Spatiotemporal dynamics of HIV propagation. J Theor Biol.

[15] Laird GM (2013). Rapid quantification of the latent reservoir for HIV-1 using viral outgrowth assay. PLoS Pathog.

[16] Rousseau CM (2004). Association of levels of HIV-1-infected breast milk cells and risk of mother-to-child transmission. J Infect Dis.

[17] Rosenbloom DIS (2015). Designing and interpreting limiting dilution assays: general principles and applications to the latent reservoir for human immunodeficiency virus-1. Open Forum Infect Dis.

[18] R Core Team (2013). R: a language and environment for statistical computing.

