# Supplementary material for: Replication competence of virions induced from CD4+ lymphocytes latently infected with HIV
Source: Retrovirology. 2019 Feb 15;16:4. doi: 10.1186/s12977-019-0466-1 (PMC6377736; doi:10.1186/s12977-019-0466-1)
Supplement: Supplementary file 1 — Additional file 1: Fig. S1. Parallel QVOA results in the presence or absence of raltegravir with CD4 lymphocytes from 4 additional subjects. Six replicates were performed for each serial threefold dilution of CD4 cells in the QVOA assay with (a–d left column) or without (a–d right column) raltegravir in the culture medium throughout the assay shown in log10 scale. Culture supernatants were assayed for HIV RNA by real time (RT)-PCR. The asterisks on the lines of the replicates without raltegravir on day 14 indicate values for each well without raltegravir that exceeded the mean plus 5 standard deviations of the 6 replicates performed with raltegravir. Columns e–h display the same data for day 14 to indicate visually that virions are induced in the presence of raltegravir (upper figure) and that amplification occurs in almost as many wells (lower figure). [file 12977_2019_466_MOESM1_ESM.pdf]

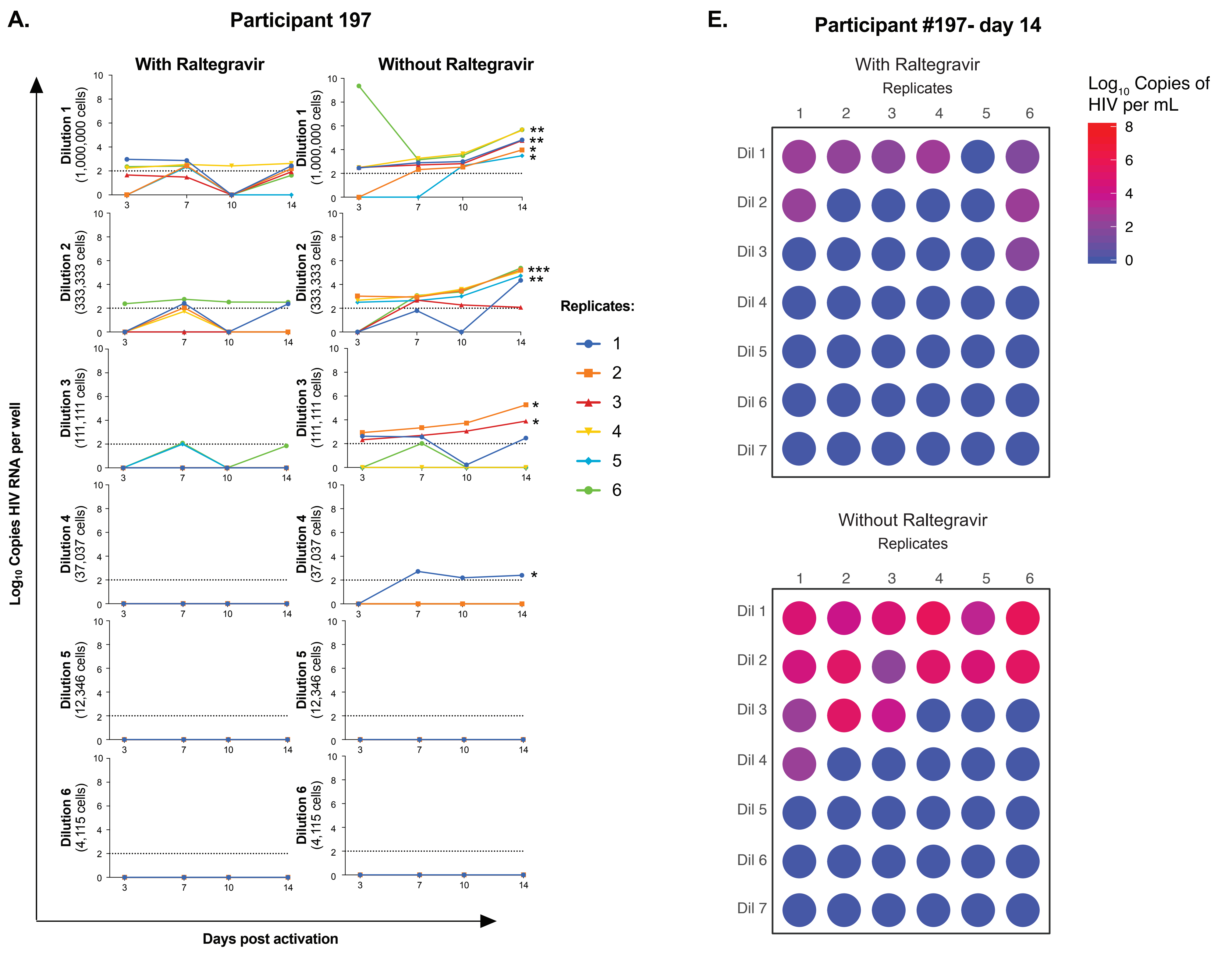
A.
Participant 197
With Raltegravir
Without Raltegravir
Dilution 1 (1,000,000 cells)
Dilution 2 (333,333 cells)
Dilution 3 (111,111 cells)
Dilution 4 (37,037 cells)
Dilution 5 (12,346 cells)
Dilution 6 (4,115 cells)
Log10 Copies HIV RNA per well
Days post activation
Replicates:
1
2
3
4
5
6
E.
Participant #197- day 14
With Raltegravir
Replicates
Without Raltegravir
Replicates
Dil 1
Dil 2
Dil 3
Dil 4
Dil 5
Dil 6
Dil 7
Log10 Copies of HIV per mL

B.

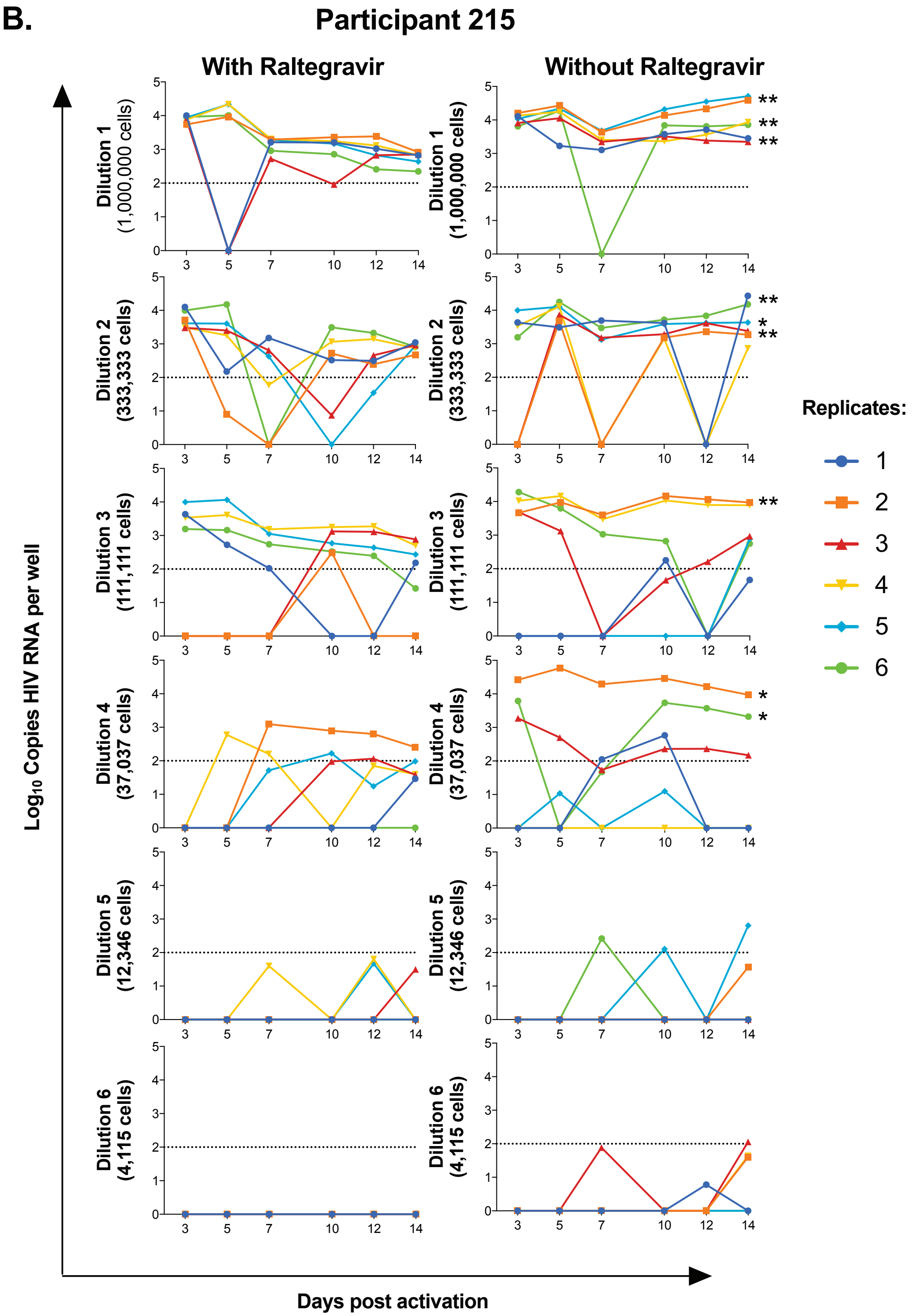

Participant 215
With Raltegravir
Without Raltegravir
Dilution 1 (1,000,000 cells)
Dilution 2 (333,333 cells)
Dilution 3 (111,111 cells)
Dilution 4 (37,037 cells)
Dilution 5 (12,346 cells)
Dilution 6 (4,115 cells)
Log10 Copies HIV RNA per well
Days post activation
**
**
**
**
*
**
**
*
*
Replicates:
1
2
3
4
5
6

F.

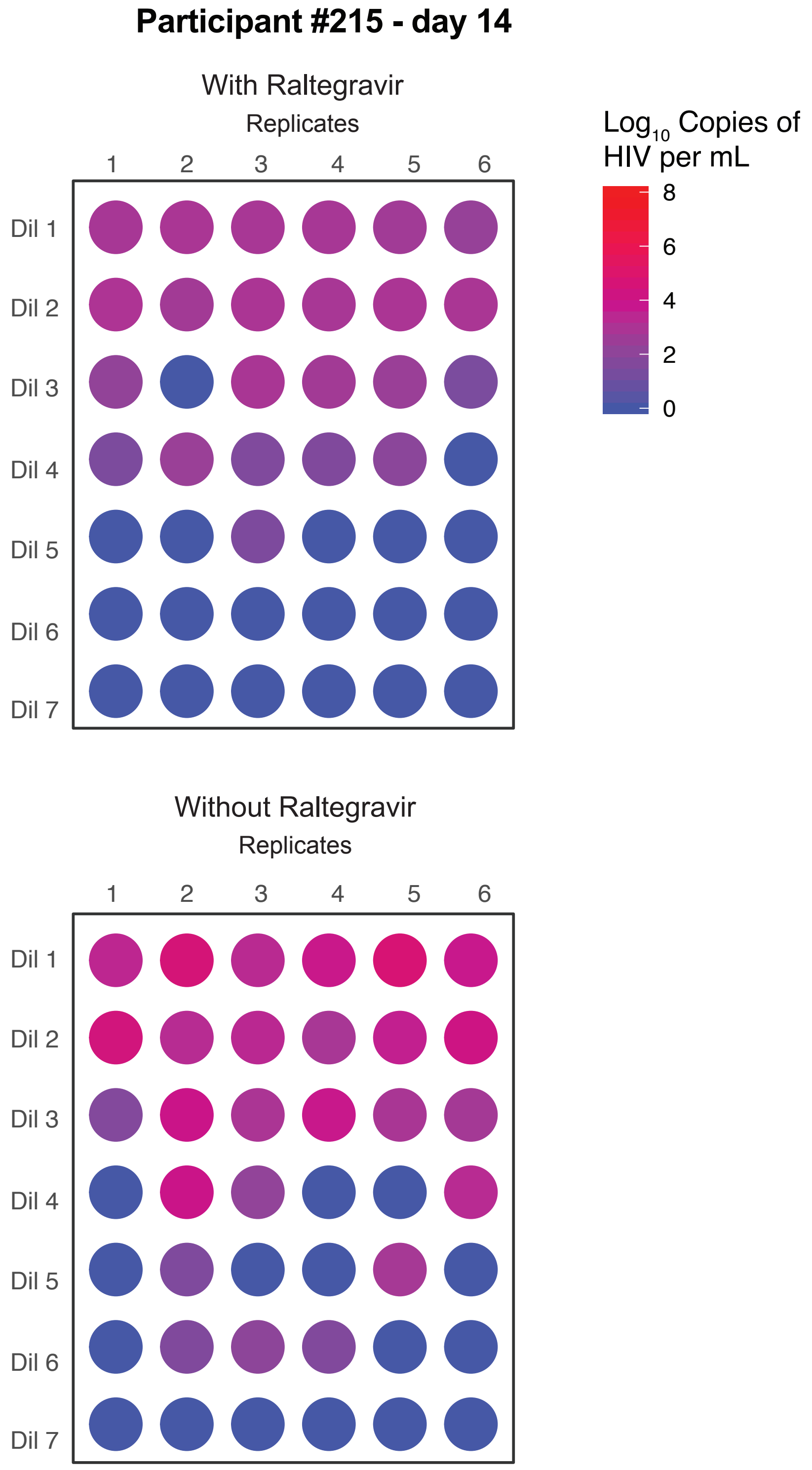

Participant #215 - day 14
With Raltegravir
Replicates
1 2 3 4 5 6
Dil 1
Dil 2
Dil 3
Dil 4
Dil 5
Dil 6
Dil 7
Without Raltegravir
Replicates
1 2 3 4 5 6
Dil 1
Dil 2
Dil 3
Dil 4
Dil 5
Dil 6
Dil 7
Log10 Copies of HIV per mL
8
6
4
2
0

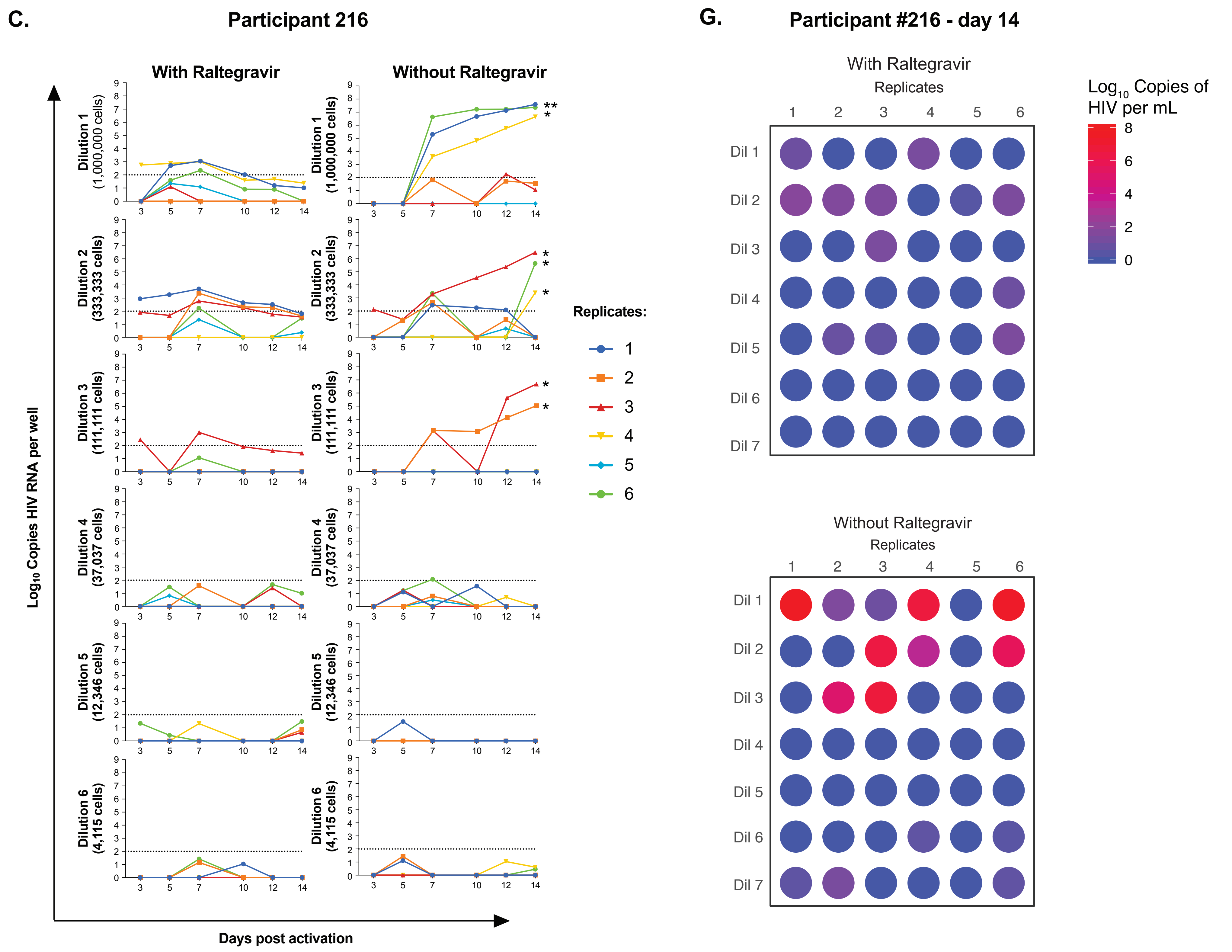
C.
Participant 216
With Raltegravir
Without Raltegravir
Log10 Copies HIV RNA per well
Dilution 1 (1,000,000 cells)
Dilution 2 (333,333 cells)
Dilution 3 (111,111 cells)
Dilution 4 (37,037 cells)
Dilution 5 (12,346 cells)
Dilution 6 (4,115 cells)
Days post activation
Replicates:
1
2
3
4
5
6
G.
Participant #216 - day 14
With Raltegravir
Replicates
Without Raltegravir
Replicates
Dil 1
Dil 2
Dil 3
Dil 4
Dil 5
Dil 6
Dil 7
Log10 Copies of HIV per mL

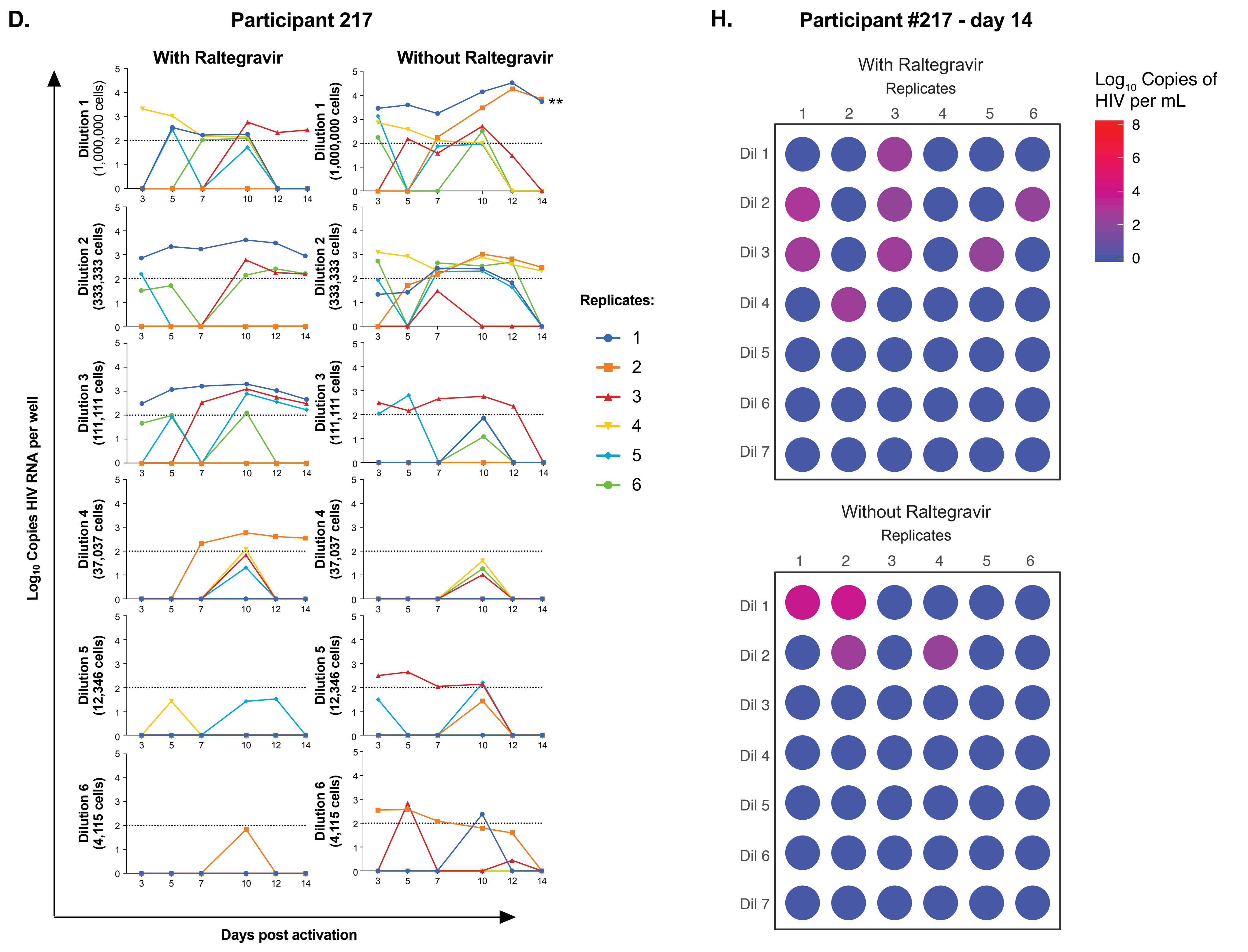
D.
Participant 217
With Raltegravir
Without Raltegravir
Dilution 1 (1,000,000 cells)
Dilution 2 (333,333 cells)
Dilution 3 (111,111 cells)
Dilution 4 (37,037 cells)
Dilution 5 (12,346 cells)
Dilution 6 (4,115 cells)
**
Log10 Copies HIV RNA per well
Days post activation
Replicates:
1
2
3
4
5
6
H.
Participant #217 - day 14
With Raltegravir
Replicates
Without Raltegravir
Replicates
Dil 1
Dil 2
Dil 3
Dil 4
Dil 5
Dil 6
Dil 7
Log10 Copies of HIV per mL
8
6
4
2
0

Additional file 1: Fig.S1. Parallel QVOA results in the presence or absence of raltegravir with CD4 lymphocytes from 4 additional subjects. Six replicates were performed for each serial 3-fold dilution of CD4 cells in the QVOA assay with (A-D left column) or without (A-D right column) raltegravir in the culture medium throughout the assay shown in log_{10} scale. Culture supernatants were assayed for HIV RNA by real time (RT)-PCR. The asterisks on the lines of the replicates without raltegravir on day 14 indicate values for each well without raltegravir that exceeded the mean plus 5 standard deviations of the 6 replicates performed with raltegravir. Columns E-H display the same data for day 14 to indicate visually that virions are induced in the presence of raltegravir (upper figure) and that amplification occurs in almost as many wells (lower figure).
